# CD26 and Cancer

**DOI:** 10.3390/cancers14215194

**Published:** 2022-10-23

**Authors:** Oscar J. Cordero

**Affiliations:** 1 Department of Biochemistry and Molecular Biology, CIBUS Building, University of Santiago de Compostela, 15782 Santiago de Compostela, Spain; oscarj.cordero@usc.es; 2 Gene Regulatory Control in Disease Group, Centre for Research in Molecular Medicine and Chronic Diseases (CiMUS), University of Santiago de Compostela, 15782 Santiago de Compostela, Spain; 3 Health Research Institute of Santiago de Compostela (IDIS), 15706 Santiago de Compostela, Spain

This Special Issue presents new knowledge on the complex behaviour of dipeptidyl peptidase 4 (DPP4, EC 3.4.14.5, also known as CD26 in the lymphoid lineage) in tumour cells and as a soluble form in many biological fluids, and on their proposed role as therapeutic targets and possible crosstalk among these CD26-related molecular participants. We hope that the assembling of these relationships may help to better understand the molecule and, importantly, to calibrate the future of those therapies.

This exoprotease is a dimer of a transmembrane glycoprotein of up to 110 kDa MW. It is expressed on a variety of cell types and tissues, mainly epithelial cells and lymphocytes (where immunologists called it CD26). The DPP4 activity, cleaving either N-terminal X-proline or X-alanine dipeptides from polypeptides, was discovered in the process of dietary protein digestion on the surface of enterocytes [1]. The membrane protein was originally identified as the adenosine deaminase binding or complexing protein (ADAbp, ADCP) [2,3,4,5,6,7]. In fact, DPP4 also acts in enzymatic-activity independent manners, as a functional receptor for ECM collagen and fibrinogen, or interacting with CD45, caveolin-1, and beta-integrins, among others. Consequently, CD26/DPP4 is considered a multifunctional or moonlighting protein [8,9,10].

CD26/DPP4 has consistently been associated with cancer since it was known as ADCP [3,4,11], when deficiencies in solubilized CD26 from total homogenates of colon, kidney, lung and liver tumours, or from cancer-derived cell lines, were described [11,12]. Its involvement in tumorigenesis has been studied extensively [13], serving as a tumour suppressor or activator depending on the tumour microenvironment.

However, its mechanisms of action are far from being completely known. First, over the years, it has been found that many biologically active polypeptides also contain the sequence cut by DPP4. One possibility is that those changes on tumour CD26 expression are associated with changes in the levels of these substrates. Probably the most well-known are hormones secreted by enteroendocrine cells of the gut that increase insulin secretion in response to the ingestion of nutrients, therefore being key modulators of glucose homeostasis: glucagon-like peptide 1 and 2 (GLP-1 and -2) and glucose-dependent insulinotropic polypeptide (GIP) [14,15,16]. The development of DPP4 inhibitors (DPP4i) to target type 2 diabetes followed. From the beginning, there have been concerns on their effects on cancer progression in diabetic patients. In this Special Issue, the group of Kanasaki review possible undesirable effects on diabetic patients [17]. Different meta-analyses of randomized clinical trials showed that DPP4i were not related to an increased risk of developing cancers compared with placebo or other anti-diabetic drugs in T2DM patients, although the follow-up period was too short for validating the impact of DPP4i on cancer incidence. However, in patients with existing breast cancer, inhibitors enhanced metastasis and chemoresistance via the increase in substrate C-X-C motif chemokine 12 (CXCL12) and the consequent induction of epithelial–mesenchymal transition in the primary tumour [17].

Ng et al. reported, in contrast, that in a cohort of colorectal patients with diabetes and treated with DPP4i, their 5-year prognosis following curative resection was significantly better than those treated with metformin. Subsequently, they compared the prognosis of post-operative CRC patients with diabetes complications and treated with DPP4i or metformin, showing that the better prognosis was associated with immune cell population features [18].

Indeed, other regulatory peptides are DPP4 substrates, such as substance P, chorionic gonadotropin, monomeric fibrin, promellitin, neuropeptides NPY, NPYY, brain natriuretic factor [9,10,19,20], and many cytokines as well as chemokines [21,22,23]. The review by De Zutter et al. [24] recapitulates current knowledge on the interplay between CD26 and chemokine activity in cancer, as chemokines are central players that can be post-translationally modified by DPP4/CD26 activity with different final effects on their functions, enhancing or reducing their activity. Different levels of tumour CD26 expression affect the dominant chemokine isoforms present in those tumour microenvironments. The most profound effect seems to be on CXCL12 (the CXCR4 ligand) and the CXCR3 ligands (CXCL 9–11), which are converted into antagonists upon truncation.

This chemokine cleavage has been reported to attenuate anticancer immunity in conjunction with the dysregulation of macrophage M1/M2 polarization (through the cleavage of MIP-1alpha, CCL3). The review by Nishina et al. [25] focuses on chemokines as promising agents to activate T cell trafficking, the importance of which is enhanced by the fact that immune checkpoint inhibitors and VEGF inhibitors, currently recognized as a combination first-line systemic treatment for some cancers, including hepatocellular cancer (HCC), influence the infiltration of T cells into tumours. The inhibition of DPP4 enzymatic activity enhances antitumor effects by preserving biologically active CXCL10 and increasing lymphocyte trafficking, and by enhancing the role of CCL11-dependent eosinophils on antitumor immunology. In this sense, DPP4i are potential adjuvant therapeutic agents, and the novel pre-clinical models presented here support their applications for ovarian cancer [26] and HCC [27], where a new antitumor activity for DPP4i, via caspase-1 activation, is described for the first time.

On the other hand, however, human CD4 T cells with high levels of CD26 expression have an enhanced chemokine receptor profile and stemness, are cytotoxic and resistant to apoptosis, and, when engineered with a chimeric antigen receptor (CAR), ablated large human tumours [28]. Subsets of CD4 CD26high T cells are Th1, Th17, hybrid Th1/Th17, or Th2 cells, as well as CD8 T cells [29]. Consequently, whether DPP4 inhibitors might hamper, (attending to the pre-clinical results in animal models) their capacity for trans-endothelial migration [30] should be studied.

Additionally, other mechanisms can be affected by the DPP4i: one collagen matrix-dependent and related to the motility capacity of both immune and tumour cells; the other related to the inhibition of metastatic traits through opposite effects to those of transforming growth factor-β1 [31].

CD26 expression has been associated with the early aggressiveness of T and B cell lymphomas and leukaemia [32]. A partial recent review of CD26 in haematological malignancies [13] is completed here with the reviews performed by the group of Lettau and Sicuranza et al. [33,34]. CD26/DPP4 expressed on T cell lymphoblastic lymphoma (T-LBL), T cell acute lymphoblastic leukaemia (T-ALL), T-ALCL (anaplastic large cell lymphoma), T-large granular lymphocyte lymphoproliferative disorder (T-LGL LPD), B-chronic lymphocytic leukaemia (B-CLL), or multiple myelomas (MMs, when associated with osteoclasts) has been associated with poor survival and associations with cell adhesion. However, in cutaneous T cell lymphoma (CTCL,) including the most frequent CTCL mycosis fungoides (MF) and Sézary syndrome (SS), the expression is lost with disease progression (associated with lower serum CD26/DPP4 levels). In this case, this loss induces the migration of SS cells by a reinforced chemoattraction [34].

At this point, it can be concluded that when the membrane expression of DPP4 is lost or altered in many cancers, its roles in cell-to-cell adhesion, through ADA or beta-integrin-1 and ECM, as mentioned above, are lost as well. However, at least in HCC, lipid peroxidation via PM DPP4 interactions with NADPH oxidase 1 (NOX1) (an interaction which can be blocked by DPP4i), which finally leads to ferroptosis, has been documented when the tumour-suppressor p53-dependent subcellular redistribution of DPP4 towards the nucleus (to act as a transcription cofactor) is lost [25]. This process is protective in cancer patients but promotes tumorigenesis. Consequently, an inducible translation of DPP4 in these cases reversed the malignant phenotype. Perhaps, tumour cells also avoid the DPP4-activity-dependent degradation of unknown autocrine growth factors in the tumour microenvironment required for the survival of those cells [22]. Some of the chemokines cited above responsible for metastatic cell migration should be removed at that moment. Additional pathways reviewed in the Special Issue show where CD26 is implicated in cancer as the ADA partner in the metabolism of nucleotides, very active in tumour tissues [33,34].

However, DPP4 participates with other cell surface proteases in malignant transformation and cancer progression by facilitating invasion [35,36,37], as was first suggested by Werb [38]. In this sense, the knowledge acquired in recent years about cancer stem cells (CSCs) is remarkable, as is the fact that subsets of CSCs expressing CD26 have been implicated in metastases of many cancers [13,31,39], including haematological (leukemic stem cells or LSCs, reviewed in [34]). In contrast to other tested antigens co-expressed on chronic myeloid leukaemia (CML) LSCs, acute myeloid leukaemia (AML) LSCs, and normal HSCs, CD26 is the only marker which is not present on CD34+/CD38− normal stem cells, and the expression levels of these cells are associated with prognosis of the disease [34].

At least in malignant pleural mesothelioma (MPM), CD26-expressing MPM cells upregulate the production of periostin, and secreted periostin enhances the migration and invasion of MPM cells [40]. Alternatively, we showed that cell-to-cell homotypic aggregation in spheroids was enhanced in the presence of CD26 [31]. Collective cell migration is more efficient than single-cell migration and highly metastatic cells form multicellular homo- and heterotypic aggregates; these features are associated with resistance to anoikis, adhesion to the endothelium and to other cells within the metastatic site, and with an increased malignant behaviour. Notably, DPP4i do not block CD26-dependent cell aggregation [31], which is coherent with the fact that fibronectin and/or ecto-ADA bind to epitopes of CD26 that do not involve its catalytic site. In addition, CD26 and polycomb BMI1, a stem cell factor, are co-expressed in LSCs [34], with BMI1 inhibition showing promising results in the treatment of chemo-resistant cancers [41].

Similarities between these stem cells and those immune system cells with higher anti-tumour abilities should be highlighted [42]. As these cells migrate, the co-expression of CD26 and chemokine-receptors on them probably changes their patterns of migration. Thus, in patients with advanced cancers, DPP4i can possibly enhance metastasis and chemoresistance via increases in CXCR4 substrate CXCL12, as has been described in breast tumours [17,25], and at the same time increase tumour infiltration by T cells. Additionally, the fact that Tregs lack CD26 expression, CD26 being a T cell co-stimulator through its interaction with caveolin-1 or ADA in antigen presenting cells (APCs), might be informative of the functions of CD26 [33,42].

Notably, in addition to DPP4 inhibitors, other experimental therapies target this protein. Anti-CD26 mAb (Belogemab) immunoliposomes loaded with venetoclax (IL-VX), an enhancer of apoptosis, successfully eliminated LSCs of CML in in vitro experiments [34]. With humanized anti-CD26 mAbs alone, some clinical trials have been started, more advanced for malignant mesotheliomas but also for other types of cancer [43,44]. Considering all the exposed aspects, some undesirable effects can be expected, and should therefore be studied, such as on immune system cells expressing CD26, or on its soluble form (sCD26, or sDPP4) [45]. The levels of “natural” or “functional” serum/plasma anti-CD26 autoantibodies are altered in some diseases, including cancer. It is not known at present how they relate to soluble or cell surface Ag [46,47].

Biological fluids contain relatively high levels of soluble CD26 (sCD26 or sDPP4) [19,48]. Altered levels of sCD26 activity have been reported in many diseases including some cancers, indicating the potential utility of this protein as a marker in the screening, monitoring, and prognosis of some cancers [19,20]. The tissue/organ sources of sCD26 seem to be the liver epithelium, as well as lymphocytes for fractions that can be altered or regulated in disease. Adipocytes (and/or macrophages in the adipose tissue) are also an important source of DPP4 in obesity; thus, DPP4 secretion from them has been proposed as a risk factor for some types of carcinogenesis, such as HCC [25]. MIP-1α is the most efficient monocyte chemoattractant after cleavage by DPP4, potentially being important for the regulation of macrophage polarization in obese states. However, in obesity-related cancers, such as colorectal cancer, DPP4 levels are impaired [19,49].

The mechanisms of the overexpression and higher secretion of sDPP4 from hepatocytes, at least in obesity and hepatocellular cancer, have been reviewed [25]. More data support shedding from cell membranes rather than secretion. Numerous proteases mediating the release of sCD26/DPP4 have been suggested—matrix metalloproteases 1 (MMP1), MMP2, MMP9, MMP10/13, and MMP14, or the serine protease kallikrein 5 (KLK5), pending on the cell tissue—which are likely differentially regulated [33]. However, CD26/DPP4 has also been detected in many types of intracellular organelles, including secretory granules from cytotoxic lymphocytes including CD8+ and CD4+ human T cells. Intracellular CD26/DPP4 is rapidly translocated to the cell surface in response to appropriate stimuli, and degranulation is accompanied by the release of proteolytically active sCD26/DPP4 [33]. It will be interesting to test whether the impairment of soluble DPP4 levels in cancers such as colorectal is associated with migration of these cytotoxic cells into the tumours.

To point out that proteolytically active CD26/DPP4 is present on plasma exosomes, including those of some carcinoma patients, derived from both tumour cells and T cells, with the capacity of ligating potential interaction partners. For instance, exosomes isolated from AML patients inhibited the colony formation of normal hematopoietic progenitor cells contributing to AML-associated cytopenia [33]. This fact may explain why sDPP4 activity and sCD26 levels detected by ELISA systems do not correlate in many cancer patients [49]. We do not know, however, the subcellular site where the sorting of CD26 to different places including the nucleus happens, or if this event is regulated. Both DPP4 activity and sCD26 levels have been proposed as biomarkers for the diagnosis and prognosis of many diseases, including cancer [19,20,24,29,44]. Despite the lack of specificity and the sex differences (not totally understood at present), possible clinical uses have been proposed [49].

Although the physiological role of sCD26 remains poorly understood, there have been interesting advances in recent years. In addition to its DPP4 activity, sCD26 binds to PAR-2 (protease-activated receptor-2), a G protein-coupled receptor, expressed in many tissues. Its effect as a chemorepellent of neutrophils, cells implicated in cancer, has been demonstrated [50]. In a rat model of HCC, high-fat diet-induced sCD26 acted cooperatively with plasma factor Xa, promoting inflammation and insulin resistance [51]. Curiously, the first activates the caveolin-1 [CAV1]-interleukin-1 receptor-associated kinase [IRAK1]-TGF-β activated kinase 1 [TAK1] pathway, whereas factor X activates the PAR2-RAF1 pathway, synergistically inducing monocyte chemokine protein 1 (MCP1) and IL-6 expression in adipose tissue macrophages [25]. CD26 (probably together with ADA) is a plasminogen receptor [52]; therefore, a careful re-evaluation of these interactions in the context of coagulation/fibrinolysis, including sCD26 Ag-anti-CD26 autoAb complexes, deserves further attention.

It has recently been shown [53] that in human-monocyte-derived macrophages, several chymotryptic serine proteases with direct links to tumorigenesis, including prostate-specific antigen (PSA), sCD26/DPP4, high-temperature requirement protein-A (HtrA), and the bacterial virulence factor subtilisin, induced indoleamine-2,3-dioxygenase (IDO1) protein expression, together with pro-inflammatory cytokine genes *IL1B* and *IL6*. The IDO1 enzymatic-activity-dependent kynurenine pathway modulates cellular activity in the brain and tolerogenesis in the immune system, being a major checkpoint in cancer development. The induction of IDO1 and cytokines by serine proteases involve the activation of NFκB, a pathway also related to the activation of PAR-2 [54].

Interestingly, TNF may act as a contributor to this activation [53], which could explain why DPP4i show a protective effect on TNF-α-induced senescence. As in a context of cancer, senescence can be considered a tumour-suppressor strategy; perhaps, low levels of sCD26 in the blood in the early stages of some cancers can be seen as pro-oncogenic in this sense.

In our study, we have demonstrated that individuals with angiodysplasia showed higher sCD26 and soluble DPP4 levels [49]. The presence in endothelial cells (also rich in CD26 [20]) of PAR-2, enhances angiogenesis on one hand [55,56] (CCL2 expression is also induced by soluble DPP4 [25]), and the well-known role of DPP4 activity in degrading pro-angiogenic chemokines, on the other [57,58,59], is a further unresolved issue probably with profound impacts in cancer treatment [24]. 

Finally, from a clinical point of view, DPP4 activity is also related to the recovery of haematopoiesis and nephroprotection after chemotherapy reviewed in [45].
